# Preferences of Older Adult Veterans With Heart Failure for Engaging With Mobile Health Technology to Support Self-care: Qualitative Interview Study Among Patients With Heart Failure and Content Analysis

**DOI:** 10.2196/41317

**Published:** 2022-12-20

**Authors:** Marva Foster, Wei Xiong, Lisa Quintiliani, Christine W Hartmann, Stephan Gaehde

**Affiliations:** 1 VA Boston Healthcare System Center for Healthcare Organization and Implementation Research Boston, MA United States; 2 Department of General Internal Medicine School of Medicine Boston University Boston, MA United States; 3 Department of Population and Public Health Sciences Keck School of Medicine University of Southern California Los Angeles, CA United States; 4 VA Bedford Healthcare System Center for Healthcare Organization and Implementation Research Bedford, MA United States; 5 Department of Public Health Zuckerberg College of Health Sciences University of Massachusetts Lowell, MA United States; 6 VA Boston Healthcare System Department of Medicine Section of Emergency Services Boston, MA United States; 7 Department of Medicine School of Medicine Boston University Boston, MA United States

**Keywords:** qualitative research, heart failure, self-care, mobile health, mobile health technology, older adults, elderly, perceptions, mhealth intervention, veteran health, mHealth technology, elderly health care, elderly self-care

## Abstract

**Background:**

Heart failure (HF) affects approximately 6.5 million adults in the United States, disproportionately afflicting older adults. Mobile health (mHealth) has emerged as a promising tool to empower older adults in HF self-care. However, little is known about the use of this approach among older adult veterans.

**Objective:**

The goal of this study was to explore which features of an app were prioritized for older adult veterans with HF.

**Methods:**

Between January and July 2021, we conducted semistructured interviews with patients with heart failure aged 65 years and older at a single facility in an integrated health care system (the Veterans Health Administration). We performed content analysis and derived themes based on the middle-range theory of chronic illness, generating findings both deductively and inductively. The qualitative questions captured data on the 3 key themes of the theory: self-care maintenance, self-care monitoring, and self-care management. Qualitative responses were analyzed using a qualitative data management platform, and descriptive statistics were used to analyze demographic data.

**Results:**

Among patients interviewed (n=9), most agreed that a smartphone app for supporting HF self-care was desirable. In addition to 3 a priori themes, we identified 7 subthemes: education on daily HF care, how often to get education on HF, support of medication adherence, dietary restriction support, goal setting for exercises, stress reduction strategies, and prompts of when to call a provider. In addition, we identified 3 inductive themes related to veteran preferences for app components: simplicity, ability to share data with caregivers, and positive framing of HF language.

**Conclusions:**

We identified educational and tracking app features that can guide the development of HF self-care for an older adult veteran population. Future research needs to be done to extend these findings and assess the feasibility of and test an app with these features.

## Introduction

Heart failure (HF) is a major health problem in the United States [1], with an estimated 6.5 million adults living with this condition. Its prevalence is highest among older adults [2-4]. Approximately 20% of all hospital discharges in older adults are associated with HF, and it is one of the major readmission diagnoses among Medicare beneficiaries [5]. Among all older adults in the United States, veterans older than 65 years represent a specifically vulnerable population, as they have a consistently higher readmission rate for HF than nonveterans [5,6]. Within the Veterans Health Administration (VHA), HF is the second most common diagnosis as well as one of the most expensive diagnoses to treat annually [6-9]. The most common reason for HF-related hospitalizations is symptom exacerbations. Engaging in self-care [10]—self-monitoring of physiologic changes and symptom recognition—is among the most critical preventative efforts to decrease HF readmission rates among patients, as early detection of potentially serious symptoms enables patients and their caregivers to intervene before hospitalization is needed [11]. HF prevention efforts, therefore, ideally target improving patients’ ability to identify and attribute meaning to early symptoms [12]. Yet, patients with HF—who are usually older persons and frequently have comorbidities—often struggle to recognize the signs of exacerbation owing to difficulty discriminating HF symptoms from other comorbidities [13,14]. They can also find it challenging to adhere to complicated medication regimens and lifestyle advice related to diet and exercise [12,15]. The use of mobile health (mHealth) technologies (eg, Fitbit and Apple Heart Study) with HF symptom–tracking features, which enable older adults to monitor changes in their conditions on a daily basis and determine when treatment is needed, may improve HF health outcomes and thereby decrease health care usage.

mHealth technology is an effective platform to support changes in health behavior (physical activity, diet, etc) because of its ease of use, consistent connectivity to information, and quick upgrades that lead to ever-increasing sophistication [16,17]. Furthermore, mHealth has the potential to be useful for symptom management among all populations including older adults because it can include behavioral prompts, reminders, illness monitoring, and self-care management programs that extend beyond clinic walls. The use of mHealth technologies among older adults is increasing [18]. Over 62% of adults aged 70 years and older use smartphones [19]. Over 30% of older adults report using a smartphone app to manage an aspect of their health [4,20]. Older veterans also report technology ownership rates and interest in using mHealth, which is in line with that of the general population [21,22]. The increased availability of mHealth tools and the increasing technological engagement of older adults offer a potentially cost-effective solution to support HF self-care.

Nevertheless, there are also a number of specific barriers for implementation in this patient population. These include older patients’ physical limitations from existing health conditions, such as sensory impairments, cognitive changes, arthritis, and vision impairments. These age- and disease-related physical limitations can inhibit the ability of older adults to use the functions of the technology and receive maximum benefit [16,23]. Other perceived challenges are the burden or workload associated with device use [24]. One suggested way to address these barriers is to involve older adults in the design process from inception through development [16,25,26].

In addition, mHealth developers should consider getting input specifically on features that will support health behavior change. Health behavior refers to any behaviors (physical activity, healthy diet, etc) that can impact a person’s physical and mental health and quality of life [27]. Successful HF health behavior change leads to better management of the disease and resultant reduction in the frequency of hospital readmissions [28,29]. Embedding health behavior change strategies into cardiovascular interventions have shown a sustainable change in self-management behaviors [30,31]. Strategies or features to support behavior change include instruction on how to perform a behavior, self-monitoring, goal setting, problem-solving, etc [32,33]. There are established national and international guidelines that support the use of behavior change features to improve cardiovascular disease, including HF [34,35]. However, key questions remain in terms of how mHealth interventions should be optimally designed for older adult veterans with HF.

A recent review of the functionalities of commercially available apps and their ability to support HF symptom monitoring and self-care management was conducted by Creber et al [36]. The authors searched 3 web-based app stores for apps that provided self-management to patients with HF. They then rated the apps using the Mobile Application Rating Scale [37] and the Heart Failure Society of America guidelines for nonpharmacologic management [38]. The authors reviewed 34 apps that met inclusion criteria and found that many apps were designed to support healthy living rather than chronic disease management and did not effectively support change in health behavior. This highlights the need for improving the ability of mHealth apps to support HF. It also opens the door to involving end users in helping identify effective and engaging mHealth interventions to improve patient self-care [39,40]. Therefore, in this preliminary study, we examined the behavior change features older adult veterans with HF would find important to include in an HF mHealth intervention.

## Methods

### Design

The study used a descriptive qualitative method [41,42]. We conducted individual, web-based, semistructured in-depth interviews among older veterans with HF. We used a qualitative content analysis approach [41] to provide a rich description of older adult veteran perspectives and preferences for a mHealth intervention to support HF self-care.

### Ethical Considerations

Ethical approval for this study was obtained from the VA Boston Healthcare System Institutional Review Board (#3216-X). The local institutional review board approved this study with a waiver of informed consent. Participants received a US $20 cash voucher for their participation. Randomly generated ID numbers were assigned to participants to ensure confidentiality. No personal identifying information was used in the audio recordings.

### Participant Sampling and Recruitment

We used purposive sampling [41] (a nonprobability selection of participants) to ensure the richness of data for this preliminary study. We recruited older veteran adults aged 70-80 years who used smartphones. Between October 2020 and May 2021, we used administrative data to obtain a list of 70 potential participants who were patients at VA Boston HF clinic. We excluded 15 patients who had cognitive impairment or a psychotic disorder diagnosis. We mailed opt-out letters to the remaining 55 patients and called potentially eligible participants who did not opt out or otherwise contact the study staff. Interested and eligible patients (n=9 for a response rate of 16%) were enrolled in the study if they (1) lived in their own house or apartment, (2) were aged 65 years or older, (3) had an HF diagnosis, (4) owned an Android or iOS platform smartphone, and (5) used any apps on their smartphone more than once in the preceding 30 days. All participants were enrolled after an introductory conversation with the researchers who explained to them the details and the purpose of the study. 

### Data Collection

Data were collected between January and July 2021. We conducted semistructured interviews using a web-based platform, WebEx (WebEx Video Communications, Inc) [43]. Participants were given the option to use the video function or call in via telephone. At the start of the interview, participants answered a short quantitative questionnaire to gather demographic information (age, race and ethnicity, level of education, and marital status), and usage of smartphone apps (see *Demographic Questionnaire* in Multimedia Appendix 1).

We developed the semistructured interview guide based on key concepts from the middle-range theory of self-care in chronic illness [10]. The theory addresses the process of maintaining health through health-promoting practices and managing illness. The 3 key concepts are self-care maintenance, self-care monitoring, and self-care management. Self-care maintenance refers to those behaviors performed to improve well-being, preserve health, or to maintain physical and emotional stability [10]. These behaviors can be related to lifestyle (eg, exercise, preparing healthy food, and coping with stress) or the medical regimen (eg, taking medication as prescribed and attending medical appointments). Self-care monitoring is a process of routine, vigilant body monitoring, surveillance, or body listening [10]. This type of monitoring is a common behavior. For example, people may monitor weight or blood pressure regularly to follow changes. Self-care management involves an evaluation of physical and emotional signs and symptoms to determine if the change is present and action is needed [10]. It requires attention to the effectiveness of a treatment and evaluation of whether that approach should be tried again in the future. The theory was chosen to guide this study because its structure focused research efforts on results that could be readily translated into practice. In addition, the interview guide included items related to preselected behavior change features (feedback on behavior, self-monitoring of the behavior, reducing negative emotions, instruction on how to perform the behavior, goal setting, social support, problem solving, and action planning) that were rated as important in the literature and our previous research [31,44-47] (see *Interview Guide* in Multimedia Appendix 1).

During the interviews, participants were asked open-ended questions as well as directed questions to explore their perceptions and preferences for receiving app-based support to help them self-manage their HF. Participants were asked to report on features that stood out as most useful or least useful, how to brand apps so they would be recognizable to those with HF, and if there were any other features that should be included. Interview prompts centered on the preselected behavior change app features. In addition, we also asked participants their perspectives on the term “heart failure.” This question was posed to determine whether the title of the diagnosis affected the veterans’ self-efficacy, as self-efficacy is an antecedent to self-care and has been found to independently predict HF self-care [10,48,49].

The HF diagnosis of all participants was confirmed through the patient’s electronic health record by confirming an HF (International Statistical Classification of Diseases and Related Problems, Tenth Revision, Clinical Modification) code. We also collected data on the type of HF, HF preserved ejection fraction, or HF reduced ejection fraction. Interviews were conducted by MF, were audio recorded, and lasted approximately 45 minutes each. They were transcribed verbatim, and transcripts were checked for accuracy.

### Data Analysis

#### Quantitative Data Analyses

Demographic data were entered into an Excel (Microsoft Corp) spreadsheet and analyzed to generate descriptive information. The participants’ ages were described using mean and SD values, and the descriptive data were summarized using frequencies and percentages.

#### Qualitative Data Analyses

We took a combined approach to analysis. We used a directed content analysis approach [42] with predetermined codes based on the middle-range theory of self-care in chronic illness followed by inductive coding to capture the accounts (experiences and views) of research participants. Interview transcripts were uploaded in the qualitative data management program NVivo (version 12; QSR International) [50]. Analysis began with 2 researchers (MF and WX) each reading 3 transcripts in their entirety to become familiar with the data. They then highlighted all text that on first impression appeared to represent the predetermined codes (see *Coding Framework* in Multimedia Appendix 1). Next, they coded all the highlighted passages using the predetermined codes. Any relevant text that could not be categorized with the initial coding scheme was given a new code. After MF and WX reached a consensus on the final coding framework, the remaining transcripts were analyzed by MF, followed by discussions and consensus generating with WX. Explanatory verbatim quotes were selected cautiously to maintain data validity and follow themes.

## Results

### Sample Characteristics

In total, 9 men opted in to participate in an interview session. Among these 9 participants, the mean age was 73.4 (range 70-80) years; the majority were White (n=7), most had completed postsecondary education (diploma or degree, n=6), and had HF with reduced ejection fraction (n=6). Five patients reported mild to moderate HF symptoms (shortness of breath with exertion and tiredness), while 4 others reported having no HF symptoms. As it was an eligibility criterion, all had either iPhones or Android-based smartphones. Most used their smartphone for email or internet at least once a day (n=8) and had experience with downloading an app (n=4), but fewer had used their phones to search for health-related information (n=3). However, the majority (n=6) reported using their phones for looking up sports information or the news and (n=5) used their phones to play games. Sociodemographic data are presented in Table 1. None of the participants had previously downloaded an app to help them manage their HF.

**Table 1:** Sociodemographic characteristics of all participants (N=9).

Demographic information			Values
Age (years), mean (SD)			73.4 (1.4)
Gender (male), n (%)			9 (100)
**Race, n (%)**			
	Black or African American	2 (22)	
	White	7 (78)	
**Ethnicity, n (%)**			
	Not Hispanic or Latino	9 (100)	
**Level of education, n (%)**			
	Less than high school	1 (11)	
	High school	2 (22)	
	Some college	2 (22)	
	Associates’ degree	3 (33)	
	Bachelor’s degree	1 (11)	
**Marital status, n (%)**			
	Married	4 (44)	
	Widowed	2 (22)	
	Divorced	2 (22)	
	Never married	1 (11)	
Living alone, n (%)			3 (33)
**Type of** **heart failure, n (%)**			
	Heart failure preserved ejection fraction	3 (33)	
	Heart failure reduced ejection fraction	6 (67)	
Had or currently used home telehealth, n (%)			3 (33)
**Ever used a heart failure self-management app, n (%)**			
	Never	9 (100)	

### Preferences for App Features and Content

Below we discuss the participants’ opinions of the app features grouped according to the 3 key concepts of the middle-range theory of chronic illness (self-care maintenance, self-care monitoring, and self-care management; Table 2). After that, we describe three iteratively developed categories related to app preference: (1) simplicity, (2) ability to engage their caregiver, and (3) positively framed language.

**Table 2:** Themes and illustrative quotes of participants.

Theme or category	Illustrative quote
Self-care maintenance	Overall, most participants thought that having an app “would be helpful.” “The app probably would be good. You could look, not only myself, [but] others that have different problems with their heart problem.” [Vet #8]
Education on daily HF^a^ care	Most participants thought that an app would help them gain knowledge about their condition. “That would probably make my life easier. You know, I’m curious about a lot of things and I would probably read it. I would look at it and then if I had [or] I thought I had something, I’d probably look it up.” [Vet #4] A few were not sure how useful an app would be: “I don’t think it would do much, to tell you the truth. Not right now, anyway.” [Vet #2]
How often to get education on HF	Participants thought the frequency of receiving education should be determined by each person: “I think the person who was getting the App should have a choice to how often they want to get the information, how often they think they need the information. Do you need it every day or should it be every day or should it be weekly or should it be done monthly, bi-weekly or whatever? I think it’s gonna be an individual choice, not just a blanket App.” [Vet #4]
Support of medication adherence	Some participants thought having an app could not only provide medication reminders but also provide education about medications: “Yes, that would be worthwhile, especially if you could use that app, [and say] ‘tell me about [a medication]’ and it would tell you.” [Vet #9] “If I had an app to do [learn about medications], the doctor would be thrilled, I'm sure.” [Vet #7] On the other hand, some participants thought medication reminders “… would get me somewhat upset.” Some who were being seen frequently by their cardiologist did not see the importance of using an app to support medication adherence. For these individuals, their frequent medical appointments were enough: “No, I doubt [the app would be useful]. I see the cardiologist like once a month.” [Vet #1]
Dietary restriction support	The need for support to adhere to dietary restrictions (limiting sodium consumption and weight management) varied based on the participant’s level of involvement with health care services (eg, home telehealth and, nutrition courses offered at the HF clinic) and whether they were the primary cooks in their homes. Those involved in services preferred less support: “I have that Telehealth thing, …and every day that thing gives me messages about what to do about medications, about your diet, you know.” [Vet #1] Those who were not the primary cooks in their homes thought it might not help:“[M]y wife watch[es] everything that I eat.” [Vet #5] Some others stated the inclusion of dietary support, “Would be helpful. Especially like I said, I’m not educated at all in nutrition.” [Vet #9]
Goal setting for exercises	When asked about setting goals for exercise, some participants mentioned that motivation played a major factor in their desire to set goals for exercise, saying things such as, “I’ve done reading, I just need the willpower to get through it,” and, “I just don't have the motivation to do it.” However, for some the desire to set goals was dependent on others: “I would welcome that [setting goals for exercise]... We started out great here, and then my wife started having problems with her back and sciatica. But I'm anticipating that that will get back on track when she completes a course of rehab and so on, but we don’t know about that, But it does inhibit my enthusiasm for getting out and around.” [Vet #7]
Stress reduction strategies (eg, meditation and breathing exercises)	When asked about how useful it would be for an app to offer methods that can be used to relax or reduce stress, most participants did not find this feature helpful: “I think it’s definitely something that the individual should … be able to choose themselves, because I don’t want to be told what do to do relax.” [Vet #4]
Self-care monitoring	Some participants thought there was utility in using an app to monitor and track symptoms: “I think that's a good idea [to be able to track symptoms].” [Vet #7] Others pointed to the issue of having to manually input data into an app as being a deterrent to self-monitoring: “Oh, that’s just more work for myself, isn’t it? I mean I would have to concentrate every day and put a) my weight in, b) my blood sugar, c) my blood pressure… So that’s 15 minutes [that] would be just fooling around with that stuff.” [Vet #4]
Self-care management	When asked about the ability to review previous symptoms, one participant remarked “… your memory plays tricks on you.” Having the ability to review previous changes in symptoms was acceptable because: “if you get the same symptom again and it happened a month ago, it would be good to find out what you did, instead of writing it down and trying to look for it through paperwork, [which is] what I did last time.” [Vet #3] Others were skeptical about how previous information could support decision decision-making and actions on future changes in symptoms: “I don’t think my phone can tell me what’s wrong with my heart. I really don’t. What kind of App can you put on your phone that tells me that my… blood pressure is running high?” [Vet #4] Almost all participants did not think that it was important to be able to contact medical help from within an app: “Well my phone already has that. It will call 9-1-1 automatically if you want.” [Vet #1]
Prompts of when to call a provider	Another important facet about self-care management is that the treatment indicated might require consultation with a health care provider. When asked if receiving prompts to notify a provider based on changes in their HF patterns that were similar to those that led to a hospitalization in the past would be helpful, almost half of the participants did not think that it would be helpful to have this feature, and one participant stated, “Actually, it'd probably scare me out of my wits, that I was gonna check out.” Yet another participant responded, “That would be helpful. To me it wouldn’t have to be a cardiologist, just somebody that’s knowledgeable.” [Vet #9]

^a^HF: heart failure.

### Inductive Themes Related to Preferences for App Features and Content

#### Simplicity

Participants expressed that “you need to make the app easy for the old people” and develop it at “common man’s level.” This preference was clearly a requirement for most:

“You know, some apps…I'll just delete…because I can't figure it out.”

Vet #3

#### Ability to Share Data With Caregivers

When asked who else should be given access their information in an app, most wanted to share data with others.

“My son and his wife, because they're the ones [who] keep an eye on me.”

Vet #8

“Let's put it this way. [As long as my wife] can access it through her computer … to retrieve it, all well and good. I’ll go along with that.”

Vet #5

#### Positively Frame HF Language

When asked about how they feel about the term “heart failure” and its effect on them, one person said, “I don’t get that it's really a failure. Anomaly might be a better, but even that's a scary word.” The term HF echoed even more negativity for another participant:

“I don't want to talk about how I'm suffering from heart failure because I'm not, you see. And somebody who is suffering from heart failure that echoes failure, failure, failure, failure in their mind. Or if it does, how have you helped them?”

Vet #4

## Discussion

### Principal Findings

In this study, we aimed to identify the mHealth design preferences of behavior change features of older adult veterans with HF to inform the design of a future mHealth intervention. Our analysis of interviews with 9 older adult Veterans demonstrated that older adults are engaged and willing to use mobile technology to support their self-care. Participants’ accounts not only identified features and characteristics important to them to support HF self-care maintenance, monitoring, and management, but also highlighted the challenge of designing mHealth for older adults with varying levels of caregiver support.

In terms of self-maintenance, veterans perceived mHealth to be helpful to gain knowledge on their HF condition but wanted the ability to choose how often they get that information. This finding adds to the literature regarding including patient perspectives in education. It has been found that educational activities that involve shared decision-making improve self-care practices [51], and user preferences have a significant impact on the use and effect of the education [52]. Participants also expressed the need for medication support to help them understand their medications, but they had mixed thoughts regarding medication reminders. Previous research has shown high satisfaction when patients receive medication reminders that are delivered at times they select and correlate with their medication schedule [53]. Gaining patients’ perspectives on the optimal number of messages and alerts is warranted to decrease psychological stress. Dietary support was a component that some participants also indicated mixed feelings about. Responses were based on the amount of caregiver support participants already had. The differing viewpoints corroborate with existing literature that encourages mHealth designers to allow users to customize mHealth for their individual case [54]. Some participants were also interested in using goal setting features for exercises and expressed the need for “willpower” to exercise. This finding reinforced the need for live coaching to facilitate exercise goal setting and attainment, in addition to app-based content (eg, self-monitoring, information modules, and exercises) [55,56]. Furthermore, the inclusion of behavior change features (ie, plan or goals and mobile diary) in mHealth correlates with a higher incidence of statistically significant outcomes [57]. Like many chronic illnesses, HF places great physical and psychological stress on patients. Participants endorsed the helpfulness of stress reduction strategies, suggesting that individuals should be able to choose the type of strategy that appeals to them. This adds to the literature that has found that stress reduction through mind/body interventions not only has benefits for healthy individuals but has many health benefits for patients in all stages of HF [58].

Monitoring of biometric measurements (weight, blood pressure, and pulse) was deemed useful by almost all participants, as long as they did not have to manually input data into an app. Their interest in self-care monitoring is in line with previous research that patients are interested in monitoring their symptoms as long as it does not intrude in their lifestyle [59]. Manual input of data has been cited as a deterrent to symptom self-care monitoring [60,61]. Our finding corroborates the findings of an existing body of literature on the importance of incorporating automatic uploading of biometric measurements to a mHealth app [57,62]. It should be noted that although Bluetooth technology (which supports automatic uploading of data) is widely available, it is not frequently used in mHealth research [57]. Participants also endorsed a feature that would allow them to evaluate past symptoms. Having a single source to record symptoms and actions taken to remedy symptoms can provide information to patients to identify worsening of HF. This finding is consistent with research that suggests the use of an electronic heart diary [63] to provide a consistent location for patients to track and review physical and psycho-emotional changes to determine if action is needed. Although receiving prompts as to when to call their provider was not seen as helpful by some participants, it was for others. This contributes to the literature on the desirability of using mHealth to guide patients to seek higher levels of care when needed [52].

In addition to feature preferences for health behavior change (reminders, monitoring, etc), veterans also had a preference for simple, easy-to-use technology. Ease of use is just one factor that can affect adherence among older adults [64]. They also wanted the ability to share data with family and caregivers. This preference was similar to previous research that explored the attitudes and preferences of older adults on warfarin therapy regarding the use of mHealth technology and health games to gain skills for self-management [65]. Another preference was the ability to customize the app once it is downloaded. Several participants indicated they wanted to have their family’s or caregiver’s support in conducting this task. These findings substantiate previous research indicating that some older adults say they need help setting up or using technology [18,66].

This study’s qualitative approach allowed for a better understanding of not only veterans’ mHealth preferences but also their feelings when told that they have HF. It is known that language can potentially transmit bias and affect the quality of care that patients subsequently receive [67,68]. Language, whether spoken or written, can also affect the attitudes of others. Our finding that participants had a negative reaction to the term “heart failure” indicates that there may be a need to reframe language surrounding a HF diagnosis.

### Limitations

This study focused solely on VHA patients, which represent a population not typically studied in mHealth contexts (eg, older adults). This may present a limit to generalizability to other populations [56,69] but also represents a needed addition to the literature on veterans. Another limitation was that most participants were White, potentially limiting the transferability of these findings to other ethnic groups. This may mean that our participants may be overly similar in certain ways, having similar backgrounds and preferences. Another potential source of bias is the small sample size. However, this study used the concept of information power rather than saturation to determine the adequacy of the sample size. Information power indicates that the more information the sample holds, relevant for the actual study, the lower number of participants is needed [70,71]. Based on our use of a specific, vulnerable population, the use of an established theory, the quality of the dialogue, and our analysis strategy, we determined the sample size to be sufficient. Although the use of a theory to guide our work was a strength of our study, there is the possibility that our use of the specific use of the middle-range theory of self-care in chronic illness may have limited the scope of the results, and future studies should take this into consideration. In addition, our focus was to examine the perceptions of features older adult veterans with HF would find important to include in a mHealth intervention; more background on demographics and other participant characteristics would have strengthened the interpretation of our findings. Finally, 3 of our participants were enrolled in or had previous experience with home telehealth. This may have caused some bias regarding their intention to use mHealth. It is also important to acknowledge that some older adults, as a result of strong personal preferences or other barriers, may never adopt mHealth [23].

### Conclusions

In conclusion, despite the proliferation of mHealth apps to manage HF [36], a dearth of information exists concerning the usage needs of older adult veterans with HF. Some needs uncovered in our study are relatively new findings, such as the potential need to positively frame HF information and the patients’ desire for their caregivers to have access to the patient’s self-care data. Other needs identified correlate with those of HF patients in general (eg, goal setting and dietary restriction). App features should facilitate addressing these needs and consider incorporating, for example, a simple interface accessible to older adults with little or no technological literacy. Future research needs to be done to extend these findings and assess the feasibility of and test an app with these features.

## Appendix

Multimedia Appendix 1 Demographic questionnaire, interview guide and coding framework.

## Abbreviations

HF: heart failure
mHealth: mobile health
VHA: Veterans Health Administration

## Data Availability

The data sets presented in this article are not readily available because they will need to be approved by the VA IRB, requiring a modification. Requests to access the datasets should be directed to MF: Marva.Foster@va.gov.

## References

[1] Benjamin EJ, Blaha MJ, Chiuve SE, Cushman M, Das SR, Deo R, de Ferranti SD, Floyd J, Fornage M, Gillespie C, Isasi CR, Jiménez MC, Jordan LC, Judd SE, Lackland D, Lichtman JH, Lisabeth L, Liu S, Longenecker CT, Mackey RH, Matsushita K, Mozaffarian D, Mussolino ME, Nasir K, Neumar RW, Palaniappan L, Pandey DK, Thiagarajan RR, Reeves MJ, Ritchey M, Rodriguez CJ, Roth GA, Rosamond WD, Sasson C, Towfighi A, Tsao CW, Turner MB, Virani SS, Voeks JH, Willey JZ, Wilkins JT, Wu JH, Alger HM, Wong SS, Muntner P, American Heart Association Statistics Committee Stroke Statistics Subcommittee (2017). Heart disease and stroke statistics—2017 update: a report from the American Heart Association. Circulation.

[2] Mozaffarian D, Benjamin EJ, Go AS, Arnett DK, Blaha MJ, Cushman M, Das SR, de Ferranti S, Després JP, Fullerton HJ, Howard VJ, Huffman MD, Isasi CR, Jiménez MC, Judd SE, Kissela BM, Lichtman JH, Lisabeth LD, Liu S, Mackey RH, Magid DJ, McGuire DK, Mohler ER, Moy CS, Muntner P, Mussolino ME, Nasir K, Neumar RW, Nichol G, Palaniappan L, Pandey DK, Reeves MJ, Rodriguez CJ, Rosamond W, Sorlie PD, Stein J, Towfighi A, Turan TN, Virani SS, Woo D, Yeh RW, Turner MB, American Heart Association Statistics Committee, Stroke Statistics Subcommittee (2016). Heart disease and stroke statistics—2016 update: a report from the American Heart Association. Circulation.

[3] Savarese G, Lund LH (2017). Global public health burden of heart failure. Card Fail Rev.

[4] Schorr EN, Gepner AD, Dolansky MA, Forman DE, Park LG, Petersen KS, Still CH, Wang TY, Wenger NK, American Heart Association Cardiovascular Disease in Older Populations Committee of the Council on Clinical CardiologyCouncil on CardiovascularStroke Nursing; Council on Arteriosclerosis‚ ThrombosisVascular Biology;Council on LifestyleCardiometabolic Health (2021). Harnessing mobile health technology for secondary cardiovascular disease prevention in older adults: a scientific statement drom the American Heart Association. Circ Cardiovasc Qual Outcomes.

[5] Gorst SL, Armitage CJ, Brownsell S, Hawley MS (2014). Home telehealth uptake and continued use among heart failure and chronic obstructive pulmonary disease patients: a systematic review. Ann Behav Med.

[6] Nuti SV, Qin L, Rumsfeld JS, Ross JS, Masoudi FA, Normand ST, Murugiah K, Bernheim SM, Suter LG, Krumholz HM (2016). Association of admission to veterans affairs hospitals vs non-veterans affairs hospitals with mortality and readmission rates among older men hospitalized with acute myocardial infarction, heart failure, or pneumonia. JAMA.

[7] Zulman DM, Pal Chee C, Wagner TH, Yoon J, Cohen DM, Holmes TH, Ritchie C, Asch SM (2015). Multimorbidity and healthcare utilisation among high-cost patients in the US Veterans Affairs Health Care System. BMJ Open.

[8] Chapko MK, Liu C, Perkins M, Li Y, Fortney JC, Maciejewski ML (2009). Equivalence of two healthcare costing methods: bottom-up and top-down. Health Econ.

[9] Krishnamurthi N, Francis J, Fihn SD, Meyer CS, Whooley MA (2018). Leading causes of cardiovascular hospitalization in 8.45 million US veterans. PLoS One.

[10] Riegel B, Jaarsma T, Strömberg A (2012). A middle-range theory of self-care of chronic illness. ANS Adv Nurs Sci.

[11] Groeneveld PW, Medvedeva EL, Walker L, Segal AG, Menno DM, Epstein AJ (2019). Association between spending and survival of chronic heart failure across veterans affairs medical centers. JAMA Netw Open.

[12] Guzman-Clark JRS, van Servellen G, Chang B, Mentes J, Hahn TJ (2013). Predictors and outcomes of early adherence to the use of a home telehealth device by older veterans with heart failure. Telemed J E Health.

[13] Yancy C, Jessup M, Bozkurt B, Butler J, Casey DE, Drazner MH, Fonarow GC, Geraci SA, Horwich T, Januzzi James L, Johnson Maryl R, Kasper Edward K, Levy Wayne C, Masoudi Frederick A, McBride Patrick E, McMurray John J V, Mitchell Judith E, Peterson Pamela N, Riegel Barbara, Sam Flora, Stevenson Lynne W, Tang W H Wilson, Tsai Emily J, Wilkoff Bruce L (2013). 2013 ACCF/AHA guideline for the management of heart failure: executive summary: a report of the American College of Cardiology Foundation/American Heart Association Task Force on practice guidelines. Circulation.

[14] Toback M, Clark N (2017). Strategies to improve self-management in heart failure patients. Contemp Nurse.

[15] Foster M (2018). A mobile application for patients with heart failure: theory- and evidence-based design and testing. Comput Inform Nurs.

[16] Masterson Creber RM, Hickey KT, Maurer MS (2016). Gerontechnologies for older patients with heart failure: what is the role of smartphones, tablets, and remote monitoring devices in improving symptom monitoring and self-care management?. Curr Cardiovasc Risk Rep.

[17] Hale K, Capra S, Bauer J (2015). A framework to assist health professionals in recommending high-quality apps for supporting chronic disease self-management: illustrative assessment of type 2 diabetes apps. JMIR Mhealth Uhealth.

[18] Anderson MP, Perrin A (2017). Tech adoption climbs among older adults. Pew Research Center.

[19] Kakulla BN (2020). 2020 Tech Trends of the 50+. AARP Research.

[20] Fox S, Duggan M Main findings. Pew Research Center.

[21] Gould CE, Loup J, Kuhn E, Beaudreau SA, Ma F, Goldstein MK, Wetherell JL, Zapata AML, Choe P, O'Hara R (2020). Technology use and preferences for mental health self-management interventions among older veterans. Int J Geriatr Psychiatry.

[22] Lipschitz J, Miller CJ, Hogan TP, Burdick KE, Lippin-Foster R, Simon SR, Burgess J (2019). Adoption of mobile apps for depression and anxiety: cross-sectional survey study on patient interest and barriers to engagement. JMIR Ment Health.

[23] Krishnaswami A, Beavers C, Dorsch MP, Dodson JA, Masterson Creber R, Kitsiou S, Goyal P, Maurer MS, Wenger NK, Croy DS, Alexander KP, Batsis JA, Turakhia MP, Forman DE, Bernacki GM, Kirkpatrick JN, Orr NM, Peterson ED, Rich MW, Freeman AM, Bhavnani SP, Innovations‚ Cardiovascular Teamthe Geriatric Cardiology Councils‚ American College of Cardiology (2020). Gerotechnology for older adults with cardiovascular diseases: JACC state-of-the-art review. J Am Coll Cardiol.

[24] Dharmarajan K, Chaudhry SI (2016). New approaches to reduce readmissions in patients with heart failure. JAMA Intern Med.

[25] Piau A, Campo E, Rumeau P, Vellas B, Nourhashémi F (2014). Aging society and gerontechnology: a solution for an independent living?. J Nutr Health Aging.

[26] Arnhold M, Quade M, Kirch W (2014). Mobile applications for diabetics: a systematic review and expert-based usability evaluation considering the special requirements of diabetes patients age 50 years or older. J Med Internet Res.

[27] Tombor I, Michie S (2017). Methods of health behavior change. Oxford Research Encyclopedia of Psychology.

[28] Paul S, Sneed NV (2004). Strategies for behavior change in patients with heart failure. Am J Crit Care.

[29] Laddu D, Ma J, Kaar J, Ozemek C, Durant RW, Campbell T, Welsh J, Turrise S (2021). Health behavior change programs in primary care and community practices for cardiovascular disease prevention and risk factor management among midlife and older adults: a scientific statement from the American Heart Association. Circulation.

[30] Valente TW (2012). Network interventions. Science.

[31] Akinosun AS, Polson R, Diaz-Skeete Y, De Kock JH, Carragher L, Leslie S, Grindle M, Gorely T (2021). Digital technology interventions for risk factor modification in patients with cardiovascular disease: systematic review and meta-analysis. JMIR Mhealth Uhealth.

[32] Suls J, Mogavero JN, Falzon L, Pescatello LS, Hennessy EA, Davidson KW (2020). Health behaviour change in cardiovascular disease prevention and management: meta-review of behaviour change techniques to affect self-regulation. Health Psychol Rev.

[33] Michie S, Richardson M, Johnston M, Abraham C, Francis J, Hardeman W, Eccles MP, Cane J, Wood CE (2013). The behavior change technique taxonomy (v1) of 93 hierarchically clustered techniques: building an international consensus for the reporting of behavior change interventions. Ann Behav Med.

[34] Piepoli MF, Hoes AW, Agewall S, Albus C, Brotons C, Catapano AL, Cooney M-T, Corrà U, Cosyns B, Deaton C, Graham I, Hall MS, Hobbs FDR, Løchen ML, Löllgen H, Marques-Vidal P, Perk J, Prescott E, Redon J, Richter DJ, Sattar N, Smulders Y, Tiberi M, van der Worp HB, van Dis I, Verschuren WMM, Binno S, ESC Scientific Document Group (2016). Eur Heart J.

[35] LeFevre ML, U.S. Preventive Services Task Force (2014). Behavioral counseling to promote a healthful diet and physical activity for cardiovascular disease prevention in adults with cardiovascular risk factors: U.S. Preventive Services Task Force Recommendation Statement. Ann Intern Med.

[36] Masterson Creber RM, Maurer MS, Reading M, Hiraldo G, Hickey KT, Iribarren S (2016). Review and analysis of existing mobile phone apps to support heart failure symptom monitoring and self-care management using the mobile application rating scale (MARS). JMIR Mhealth Uhealth.

[37] Stoyanov SR, Hides L, Kavanagh DJ, Zelenko O, Tjondronegoro D, Mani M (2015). Mobile app rating scale: a new tool for assessing the quality of health mobile apps. JMIR Mhealth Uhealth.

[38] Lindenfeld J, Albert NM, Boehmer JP, Collins SP, Ezekowitz JA, Givertz MM, Katz SD, Klapholz M, Moser DK, Rogers JG, Starling RC, Stevenson William G, Tang W H Wilson, Teerlink John R, Walsh Mary N, Heart Failure Society of America (2010). HFSA 2010 comprehensive heart failure practice guideline. J Card Fail.

[39] Milne-Ives M, Lam C, De Cock C, Van Velthoven MH, Meinert E (2020). Mobile apps for health behavior change in physical activity, diet, drug and alcohol use, and mental health: systematic review. JMIR Mhealth Uhealth.

[40] Dennison L, Morrison L, Conway G, Yardley L (2013). Opportunities and challenges for smartphone applications in supporting health behavior change: qualitative study. J Med Internet Res.

[41] Patton M (2015). Qualitative Research and Evaluation Methods, 4th ed.

[42] Hsieh H, Shannon SE (2005). Three approaches to qualitative content analysis. Qual Health Res.

[43] (2022). Webex by Cisco. Cisco.

[44] Quintiliani LM, Foster M, Oshry LJ (2019). Preferences of mHealth app features for weight management among breast cancer survivors from underserved populations. Psychooncology.

[45] Patterson K, Davey R, Keegan R, Kunstler B, Woodward A, Freene N (2022). Behaviour change techniques in cardiovascular disease smartphone apps to improve physical activity and sedentary behaviour: systematic review and meta-regression. Int J Behav Nutr Phys Act.

[46] Duff OM, Walsh DM, Furlong BA, O'Connor NE, Moran KA, Woods CB (2017). Behavior change techniques in physical activity eHealth interventions for people with cardiovascular disease: systematic review. J Med Internet Res.

[47] Riegel B, Westland H, Iovino P, Barelds I, Bruins Slot J, Stawnychy MA, Osokpo O, Tarbi E, Trappenburg JC, Vellone E, Strömberg Anna, Jaarsma T (2021). Characteristics of self-care interventions for patients with a chronic condition: a scoping review. Int J Nurs Stud.

[48] Schweitzer RD, Head K, Dwyer JW (2007). Psychological factors and treatment adherence behavior in patients with chronic heart failure. J Cardiovasc Nurs.

[49] Schnell-Hoehn K, Naimark BJ, Tate Rbert B (2009). Determinants of self-care behaviors in community-dwelling patients with heart failure. J Cardiovasc Nurs.

[50] (2022). Powerful research simplified. QSR International.

[51] Athilingam P, Osorio RE, Kaplan H, Oliver D, O'neachtain T, Rogal PJ (2016). Embedding patient education in mobile platform for patients with heart failure: theory-based development and beta testing. Comput Inform Nurs.

[52] Allida S, Du H, Xu X, Prichard R, Chang S, Hickman LD, Davidson PM, Inglis SC (2020). mHealth education interventions in heart failure. Cochrane Database Syst Rev.

[53] Park LG, Howie-Esquivel J, Chung ML, Dracup K (2014). A text messaging intervention to promote medication adherence for patients with coronary heart disease: a randomized controlled trial. Patient Educ Couns.

[54] Alnosayan N, Chatterjee S, Alluhaidan A, Lee E, Houston Feenstra L (2017). Design and usability of a heart failure mHealth system: a pilot study. JMIR Hum Factors.

[55] Park LG, Ng F, K Shim J, Elnaggar A, Villero O (2020). Perceptions and experiences of using mobile technology for medication adherence among older adults with coronary heart disease: a qualitative study. Digit Health.

[56] Breland JY, Agha K, Mohankumar R (2021). Adoption and appropriateness of mHealth for weight management in the real world: a qualitative investigation of patient perspectives. JMIR Form Res.

[57] Donevant S, Estrada RD, Culley JM, Habing B, Adams SA (2018). Exploring app features with outcomes in mHealth studies involving chronic respiratory diseases, diabetes, and hypertension: a targeted exploration of the literature. J Am Med Inform Assoc.

[58] Aggarwal M, Bozkurt B, Panjrath G, Aggarwal B, Ostfeld RJ, Barnard ND, Gaggin H, Freeman AM, Allen K, Madan S, Massera D, Litwin SE, American College of Cardiology’s NutritionLifestyle Committee of the Prevention of Cardiovascular Disease Council (2018). Lifestyle modifications for preventing and treating heart failure. J Am Coll Cardiol.

[59] Sohn A, Speier W, Lan E, Aoki K, Fonarow G, Ong M, Arnold C (2019). Assessment of heart failure patients' interest in mobile health apps for self-care: survey study. JMIR Cardiol.

[60] Lu X, Yang H, Xia X, Lu X, Lin J, Liu F, Gu D (2019). Interactive mobile health intervention and blood pressure management in adults. Hypertension.

[61] Alessa T, Abdi S, Hawley MS, de Witte L (2018). Mobile apps to support the self-management of hypertension: systematic review of effectiveness, usability, and user satisfaction. JMIR Mhealth Uhealth.

[62] Earle KA, Istepanian RS, Zitouni K, Sungoor A, Tang B (2010). Mobile telemonitoring for achieving tighter targets of blood pressure control in patients with complicated diabetes: a pilot study. Diabetes Technol Ther.

[63] Arulnathan A, Vaaheesan S, Denecke K (2019). A mobile application for self-monitoring for patients with heart failure. Stud Health Technol Inform.

[64] Cajita M, Gleason KT, Han H-R (2016). A systematic review of mHealth-based heart failure interventions. J Cardiovasc Nurs.

[65] Lee J, Nguyen AL, Berg J, Amin A, Bachman M, Guo Y, Evangelista L (2014). Attitudes and preferences on the use of mobile health technology and health games for self-management: interviews with older adults on anticoagulation therapy. JMIR Mhealth Uhealth.

[66] Lefler LL, Rhoads SJ, Harris M, Funderburg AE, Lubin SA, Martel ID, Faulkner JL, Rooker JL, Bell DK, Marshall H, Beverly CJ (2018). Evaluating the use of mobile health technology in older adults with heart failure: mixed-methods study. JMIR Aging.

[67] Park J, Saha S, Chee B, Taylor J, Beach MC (2021). Physician use of stigmatizing language in patient medical records. JAMA Netw Open.

[68] P Goddu A, O'Conor KJ, Lanzkron S, Saheed MO, Saha S, Peek ME, Haywood C, Beach MC (2018). Do words matter? Stigmatizing language and the transmission of bias in the medical record. J Gen Intern Med.

[69] Eibner CK, Krull H, Brown KM, Cefalu M, Mulcahy AW, Pollard MS, Shetty K Current and projected characteristics and unique health care needs of the patient population served by the Department of Veterans Affairs. RAND.

[70] Malterud K, Siersma VD, Guassora AD (2016). Sample size in qualitative interview studies: guided by information power. Qual Health Res.

[71] Thorne S (2020). Untangling the misleading message around saturation in qualitative nursing studies. Nurse Author Editor.

