# Supplementary material for: Preferences of Older Adult Veterans With Heart Failure for Engaging With Mobile Health Technology to Support Self-care: Qualitative Interview Study Among Patients With Heart Failure and Content Analysis
Source: JMIR Form Res. 2022 Dec 20;6(12):e41317. doi: 10.2196/41317 (PMC9812271; doi:10.2196/41317)
Supplement: Multimedia Appendix 1 [file formative_v6i12e41317_app1.docx]

**Appendix 1**

Demographic Questionnaire

Thank you for agreeing to join the study. The following questions asks about you and your smartphone use. Please answer the questions, keeping in mind that there are no right or wrong answers. Your answers are completely private and will not be shared with anyone. Your participation is completely voluntary. You can skip any question that you do not want to answer. Thank you for your time and for helping with this project.

Participant ID#: ___________

Date: ___________

**Section I: Demographic**

1. What are the best times and numbers for reaching you?

Home: (___)____________________ Times__________________

Cell: (___)____________________ Times__________________

Work: (___)____________________ Times__________________

1. What is your age? ________ years
2. What is your gender? (CIRCLE ONE) Female Male
3. What is the highest level of education you have completed?
   1. 8^th^ grade or less
   2. Grades 9-11
   3. High school graduate
   4. Technical/business school
   5. Some college
   6. College graduate
   7. Graduate school
4. How would you describe your race/ethnicity?
   1. Caucasian
   2. African American
   3. Hispanic
   4. Asian
   5. Others (Specify:_____________________)
5. Would you say your health in general is:
   1. Excellent b. Very good c. Good d. Fair e. Poor
6. Which of the following best describes your current employment status?
   1. Employed full-time b. Unemployed c. Homemaker

d. Retired e. Employed part-time

1. Marital Status: (CIRCLE ONE)

a. never b. married c. divorced d. widowed e. separated f. engaged g. living together

**Section II: Smartphone use**

1. Do you use your smartphone to find information or complete task on the internet?
   1. No
   2. Not sure
   3. Yes
2. Please indicate which of the following online activities you have completed or attempted to complete in the last 30-days on your mobile phone.
   - Buy or make a reservation for travel
   - Email or Facebook
   - Financial/stock trading
   - Job search
   - Look up a recipe
   - Look for health/medical info
   - Look for info on a hobby or interest
   - News/weather/sports/blog
   - Online banking or bill paying
   - Online classified ads or auctions [e.g. Ebay]
   - Shopping
   - Visit a local, state or federal government website
   - Web searches
   - Look for directions
   - Playing games
   - None of the above
   - Other:
3. Have you ever used a phone application aka app to self-manage your heart failure?
   1. Yes
   2. No
4. In the last 30 days, how many times have you used an app?
   1. None
   2. Once
   3. More than once
5. Which statement best describes the reason that you have used an app:
   1. Get information about health
   2. Track meal intake
   3. Track exercise activity
   4. Get directions to a place
   5. Other
6. Have you ever been a participant of home telehealth services? If so, indicate whether you are currently participating or have been in the past.
   1. Current user
   2. Past user

**Appendix 2**

Interview Guide

I’d like to begin by asking you to please tell me a little bit about your experience with heart failure?

a. What is it like having heart failure?

b. What types of symptoms do you have?

c. How does it affect your life?

d. How do you handle having heart failure?

e. What have you tried to help you take better care of your heart failure?

**Key Concept 1** –Self-care maintenance

What are your thoughts about using a phone app to get information about heart failure?

Can you tell me your thoughts about using a phone app to learn about your medications?

What do you think about getting reminders to take your medications, doctor visits?

Would you like the phone application to explain any treatments that are prescribed for you?

Life is filled with stress, what do you think about the phone app offering you healthy ways to cope with stress?

What are do you think about the phone app helping you set goals for daily fluid intake, exercise or salt intake?

**Key Concept 2**--- Self-care monitoring

You mentioned earlier about your symptoms

What if a phone application could keep track of those symptoms?

What are your thoughts about keeping track your weight or blood pressure every day?

How would using a phone application to store that information in one place make it easier for you?

How about getting feedback on how well you are doing?

**Key Concept 3** - Self-care management

How would you like it if the phone application could remind you of the last time, you had symptoms and what you did to fix it? For example, reduced your salt intake or take an extra water pill, etc.

How about if the phone application could teach you the signs and symptoms of worsening heart failure and when to call your healthcare provider?

How important would it be to you that you had the ability to push in button in the application that would call your healthcare provider or 911 if needed?

Would you like anyone else to have access to the info in the app besides yourself (e.g., caregiver)?

What do you think about the term heart failure? Is there another term you think we should use?

**Final Wrap Up Question**

Before we wrap things up, is there anything else you think we should know in order to best design a mobile app about heart failure?

**Appendix 3**

Qualitative coding framework

| 1. **1. Deductive Codes** | **Subcodes** | **Definition/Description** | **Example** |
| --- | --- | --- | --- |
| A. Self-care maintenance codes | A1. Education on daily heart failure care | A1. Knowledge on changes in diet, lifestyle, medicines, and other treatments that can protect the heart | A1. Enhance knowledge on daily management of heart failure |
|  | A2. How often to get education on heart failure | A2. Frequency of receiving heart failure self-care education | A2. Ability to receive individualized preferences for the receipt of heart failure education |
|  | A3. Support of medication adherence | A3. Strategies to reduce barriers such as forgetfulness, motivation, fear of side effects, false beliefs about disease or treatment, and lack of understanding about medication purpose | A3. Provision of reminders (push notifications, text messages), refill reminders, teaching on medications (effects and side effects) and medication diaries |
|  | A4. Dietary restriction support | A4. Strategies to reduce the salt content in diet and fluids | A4. Encourage reading of food labels and how to choose foods that are low in salt |
|  | A5. Goal setting for exercises | A5. Setting achievable goals to help improve physical fitness. | A5. Track physical activity and reduce sedentary behaviors, e.g., television viewing |
|  | A6. Stress reduction strategies (e.g., meditation, breathing exercises) | A6. Use of healthy means to reduce psychosocial stress | A6. Suggest healthy means to reduce stress such as gardening, walking, meditation, and deep breathing. |
| B. Self-care monitoring codes |  | B. Process of routine, vigilant body monitoring, surveillance, or body listening | B. Daily monitoring of changes in breathing, weight, new or worse swelling and changes in the ability to do everyday things in a symptom diary |
| C. Self-care management codes | C. Prompts of when to call a provider | C. Indicators as to when to seek help for signs of worsening heart failure | C. Increased awareness of weight gain, worsened cough and/or increased or new shortness of breath |
| **2. Inductive Codes** | **Subcodes** | **Definition/Description** | **Example** |
| D. Simplicity |  | D. Aesthetic or logically understandable and simple interface | D. Easy access to information, ease of navigation and simple and consistent style |
| E. Ability to share data with caregivers |  | E. Sharing information between patient and caregiver | E. Tailor mobile health allow caregivers access to patient health data |
| F. Positively frame HF language |  | F. The use of positive language to reduce negative patient attitudes toward their condition | F. Ways in which medical diagnosis nomenclature can foster negative or positive attitudes toward self-care |
